# Supplementary material for: Impaired humoral immunity to BQ.1.1 in convalescent and vaccinated patients
Source: Nat Commun. 2023 May 19;14:2835. doi: 10.1038/s41467-023-38127-y (PMC10199003; doi:10.1038/s41467-023-38127-y)
Supplement: Supplementary file 1 — Supplementary information [file 41467_2023_38127_MOESM1_ESM.pdf]

Supplementary information

Impaired humoral immunity to BQ.1.1 in convalescent and vaccinated patients

**Supplementary Figure 1**	Recruitment of emergency department patients
**Supplementary Figure 2**	CONSORT flow diagram
**Supplementary Figure 3**	SARS-CoV-2 antibody prevalence in emergency department patients (additional features)
**Supplementary Figure 4**	Sub-sampling for determination of BQ1.1 serum neutralization (age and sex)
**Supplementary Figure 5**	Sub-sampling for determination of BQ1.1 serum neutralization (S-IgG and ID_{50})
**Supplementary Table 1**	Study demographics, clinical information, and SARS-CoV-2 immune status

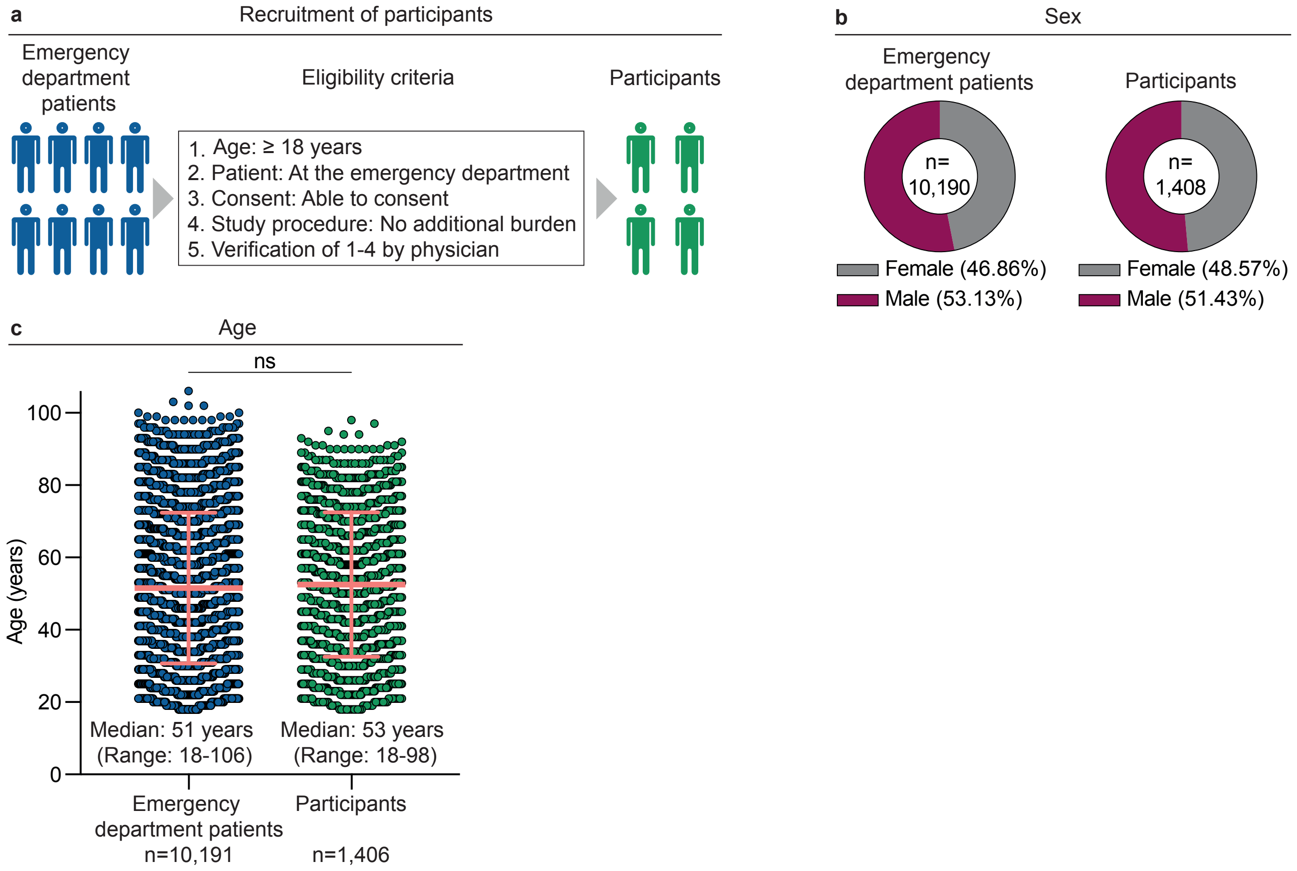

Supplementary Figure 1: Recruitment of emergency department patients

(a) Illustration depicting eligibility criteria for participation.

(b) Pie charts illustrating sex distribution of study population and sample. Sex of 1 patient and 3 participants was not available. Two-sided Fisher´s exact test (p=0.231) was performed for statistical analyses.

(c) Dot plot illustrating age distribution of study population and sample. Age of 5 participants was not available. Median age is indicated by horizontal line, standard deviation is indicated by error bars. Two-sided Mann-Whitney test was performed for statistical analyses. Ns indicates a p-value < 0.05.

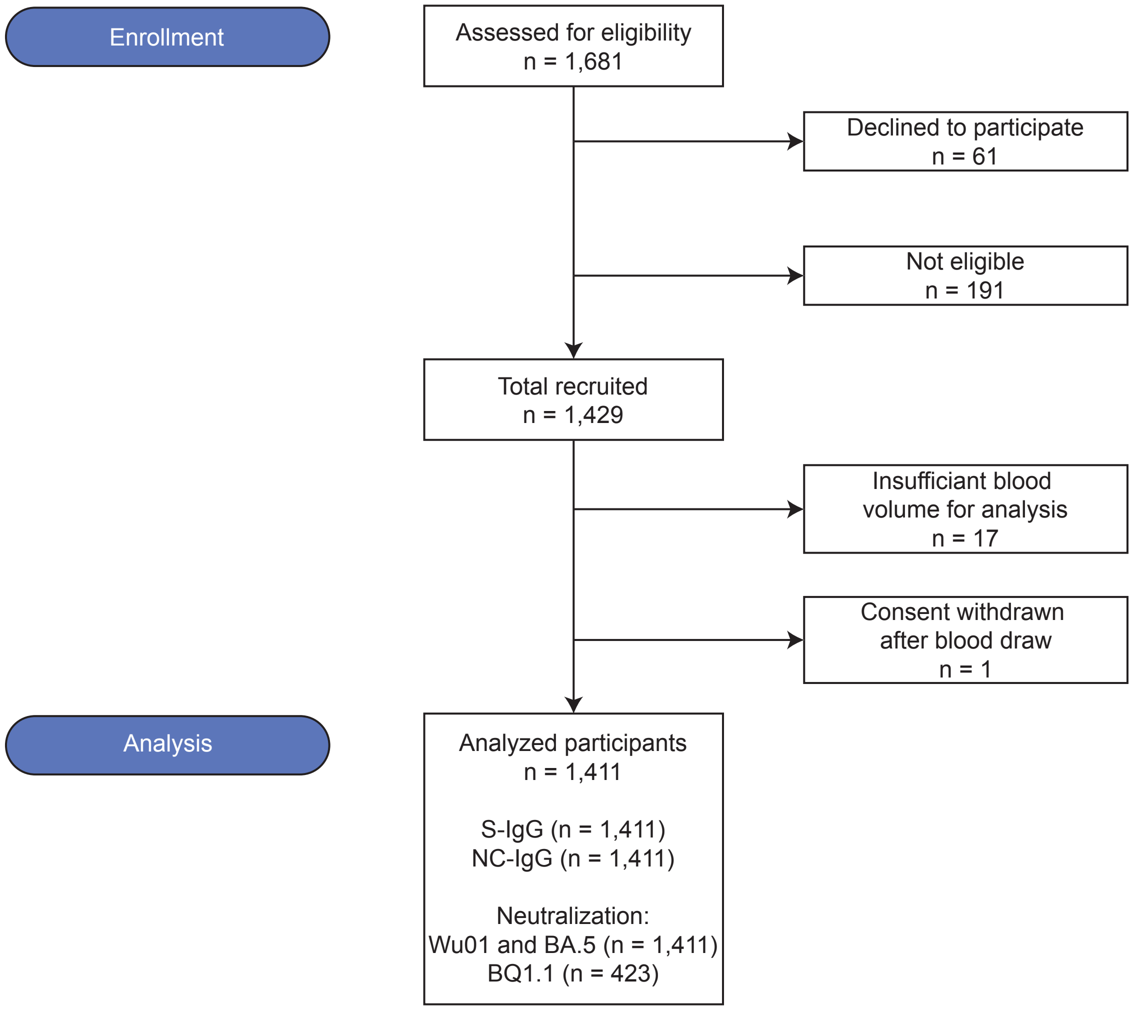

Supplementary Figure 2: CONSORT flow diagram

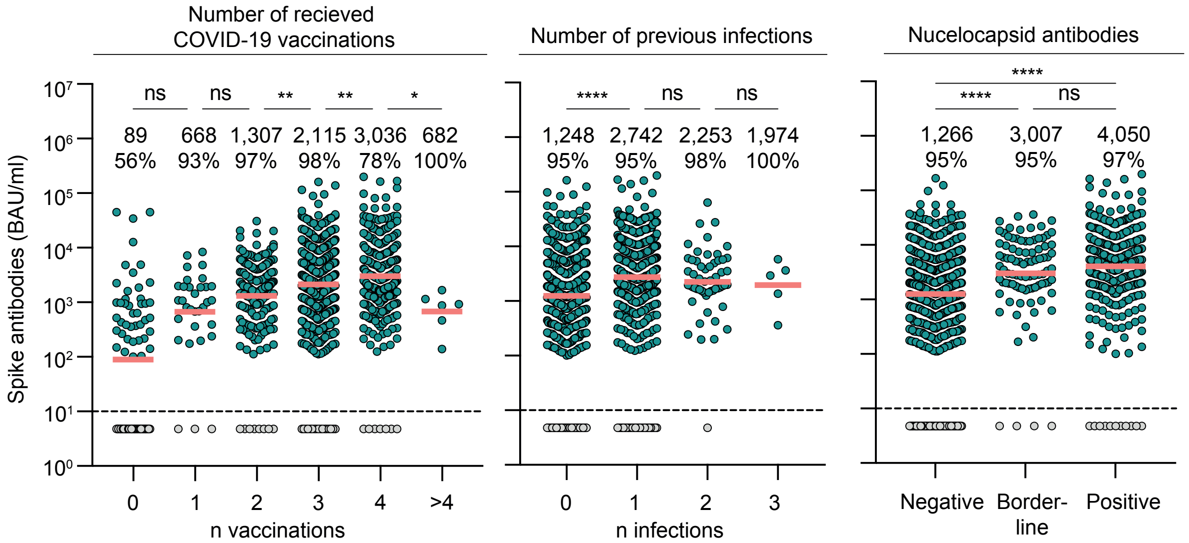

Supplementary Figure 3: SARS-CoV-2 antibody prevalence in emergency room patients (additional features)

Dot plots depicting S-IgG BAU/ml values of all participants (n = 1,411), subdivided based on number of reported vaccinations, number of reported infections, and NC-IgG detection. Dotted lines represent the limit of detection (33.8 BAU/ml). Geometric means are indicated by horizontal red lines and listed in each plot over total fractions of participants with detectable Spike IgG. Two-sided Kruskal-Wallis-tests and Dunn's multiple comparisons tests were performed for statistical analyses. Ns, *, **, ***, and **** represent p-values ≥ 0.05, < 0.05, ≤ 0.01, ≤ 0.001, and ≤ 0.0001, respectively .

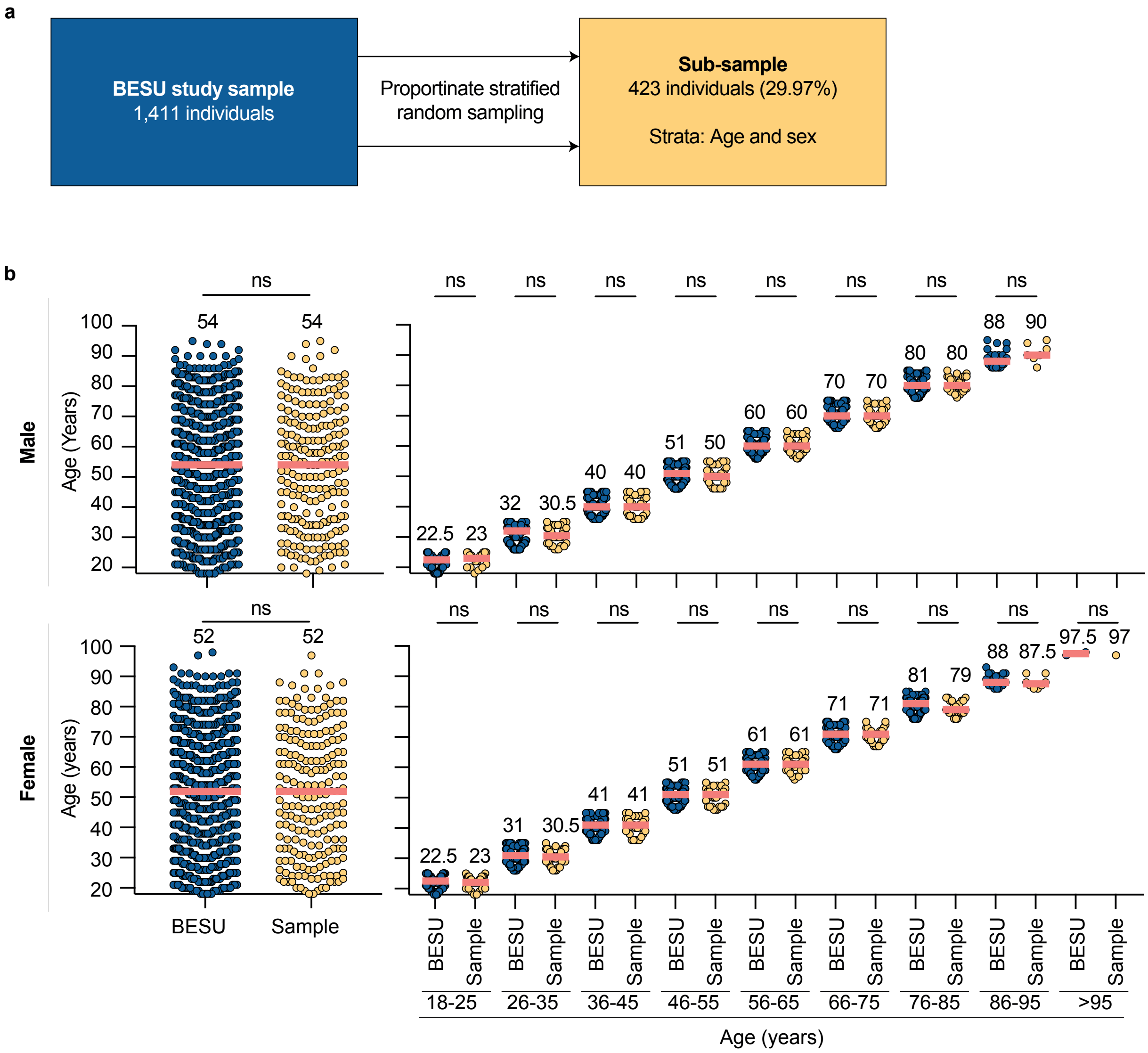

Supplementary Figure 4: Sub-sampling for determination of BQ1.1 serum neutralization (Age and sex)

(a) Illustration depicting statistical procedure for sub-sampling. Proportionate stratified random sampling was conducted to draw a representative sub-sample (n = 423).

(b) Dot plots depicting age distributions of all enrolled participants (BESU study sample; n=1,411) and the sub-sample (n = 423), subdivided by sex and age-strata. Median ages are indicated by horizontal red lines and given above the dots.
Two-sided Mann-Whitney tests and Kruskal-Wallis tests were performed for statistical analyses.
Ns represents p-values > 0.05.

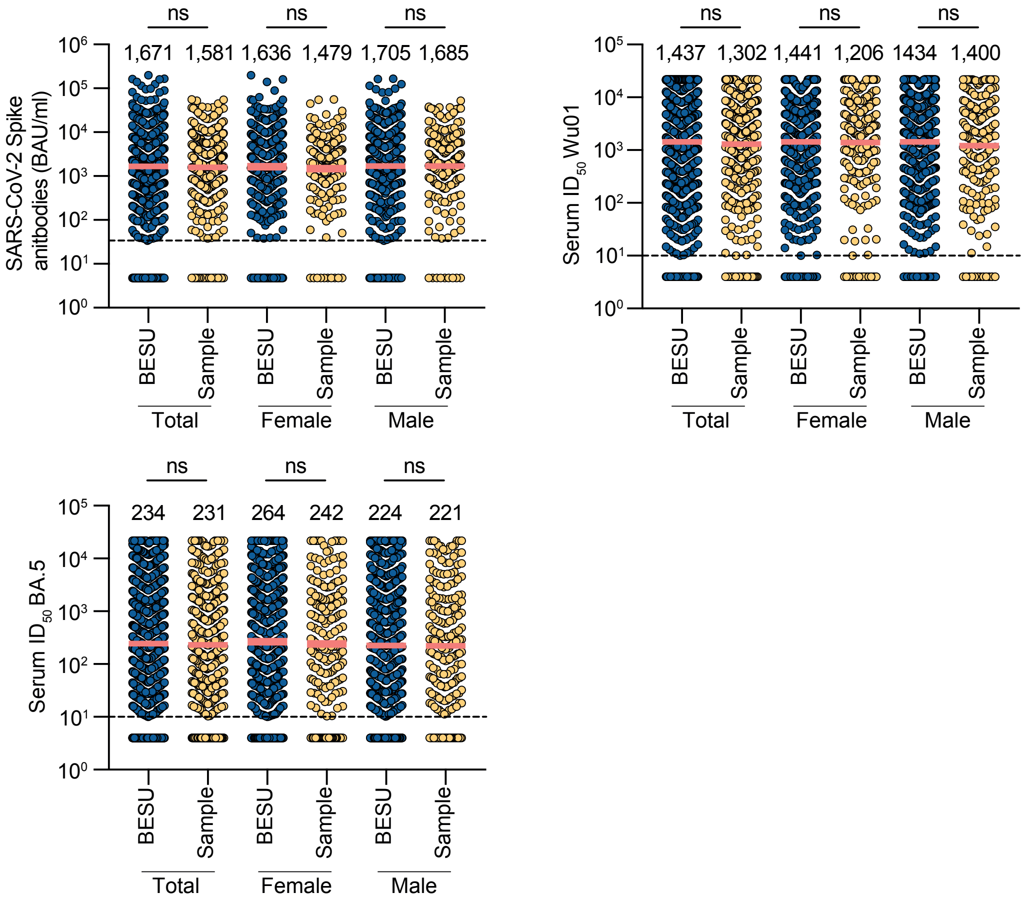

Supplementary Figure 5: Sub-sampling for determination of BQ1.1 serum neutralization (S-IgG and ID_{50})

Dot plots depicting S-IgG values (BAU/ml) and serum neutralization (ID50) of the entire study population (n = 1,411) and the sub-sample (n = 423) stratified by sex. Geometric means are indicated by horizontal red lines and given above the dots. Two-sided Mann-Whitney tests and Kruskal-Wallis tests were performed for statistical analyses.
Ns represents p-values > 0.05**.**

Supplementary Table 1: Study demographics, clinical information, and SARS-CoV-2 immune status

Gender	Male		Female		Unknown	
	(n)	*(%)*	*(n)*	*(%)*	*(n)*	*(%)*
	684	48,48	724	51,31	3	0,21

Age	Median	Range
	(years)	(years)
	53	18-89

Pre-conditions	No		Yes		Unknown	
	(n)	*(%)*	*(n)*	*(%)*	*(n)*	*(%)*
	482	34,16	906	64,21	23	1,63

Drug immunosuppression	No		Yes		Unknown	
	(n)	*(%)*	*(n)*	*(%)*	*(n)*	*(%)*
	1186	84,05	192	13,61	33	2,34

COVID-19 vaccination	Vaccination status	*(n)*	*(%)*	Time since last vaccination *Median (months)*	*Range (months)*
	No vaccination	74	5,24	NA	NA
	1 vaccination	32	2,27	11,17	6,1-19,7
	2 vaccinations	197	13,96	10,9	1,5-28,4
	3 vaccinations	766	54,29	8,733	0-20
	4 vaccinations	331	23,46	4,933	0-19,7
	>4 vaccinations	6	0,43	5,7	1,7-10,6
	Unknown	5	0,35	NA	NA

SARS-CoV-2 infections	Infection status	*(n)*	*(%)*	Time since last infection *Median (months)*	*Range (months)*
	No infection	766	54,29	NA	NA
	1 infection	579	41,03	5,30	0-32,6
	2 infections	55	3,90	4,40	0,4-19,3
	3 infections	5	0,35	5,60	2,9-7,4
	Unknown	6	0,43	NA	NA

NA= Not applicable
