# Supplementary material for: Impaired humoral immunity to BQ.1.1 in convalescent and vaccinated patients
Source: Nat Commun. 2023 May 19;14:2835. doi: 10.1038/s41467-023-38127-y (PMC10199003; doi:10.1038/s41467-023-38127-y)
Supplement: Supplementary file 3 — Reporting Summary [file 41467_2023_38127_MOESM3_ESM.pdf]

nature portfolio

Corresponding author(s): Florian Klein

Last updated by author(s): 04.04.2023

Reporting Summary

Nature Portfolio wishes to improve the reproducibility of the work that we publish. This form provides structure for consistency and transparency in reporting. For further information on Nature Portfolio policies, see our Editorial Policies and the Editorial Policy Checklist.

Statistics

For all statistical analyses, confirm that the following items are present in the figure legend, table legend, main text, or Methods section.

n/a	Confirmed	
☐	☒	The exact sample size (*n*) for each experimental group/condition, given as a discrete number and unit of measurement
☐	☒	A statement on whether measurements were taken from distinct samples or whether the same sample was measured repeatedly
☐	☒	The statistical test(s) used AND whether they are one- or two-sided<br>*Only common tests should be described solely by name; describe more complex techniques in the Methods section.*
☐	☒	A description of all covariates tested
☒	☐	A description of any assumptions or corrections, such as tests of normality and adjustment for multiple comparisons
☐	☒	A full description of the statistical parameters including central tendency (e.g. means) or other basic estimates (e.g. regression coefficient) AND variation (e.g. standard deviation) or associated estimates of uncertainty (e.g. confidence intervals)
☐	☒	For null hypothesis testing, the test statistic (e.g. *F*, *t*, *r*) with confidence intervals, effect sizes, degrees of freedom and *P* value noted<br>*Give P values as exact values whenever suitable.*
☒	☐	For Bayesian analysis, information on the choice of priors and Markov chain Monte Carlo settings
☒	☐	For hierarchical and complex designs, identification of the appropriate level for tests and full reporting of outcomes
☐	☒	Estimates of effect sizes (e.g. Cohen's *d*, Pearson's *r*), indicating how they were calculated

Our web collection on statistics for biologists contains articles on many of the points above.

Software and code

Policy information about availability of computer code

Data collection	Data collection was done using Microsoft Excel for Mac (v.14.7.3.).
Data analysis	Maps of Germany and North Rhine-Westphalia were designed with the iMapU tool provided by iExcelU. Data Analysis was performed using Microsoft Excel for Mac (v.14.7.3.), Prism 9.0 (GraphPad), and RStudio (lmtest: 0.9-40, bnlearn: 4.8.1 stats: 4.2.2). Data analysis is provided under this link: https://doi.org/10.5281/zenodo.7466593

For manuscripts utilizing custom algorithms or software that are central to the research but not yet described in published literature, software must be made available to editors and reviewers. We strongly encourage code deposition in a community repository (e.g. GitHub). See the Nature Portfolio guidelines for submitting code & software for further information.

Data

Policy information about availability of data

All manuscripts must include a data availability statement. This statement should provide the following information, where applicable:

- Accession codes, unique identifiers, or web links for publicly available datasets
- A description of any restrictions on data availability
- For clinical datasets or third party data, please ensure that the statement adheres to our policy

A Source Data file is provided with this manuscript. Raw data reported in this paper will be shared by the lead contact upon reasonable request. SARS-CoV-2 variant

proportions were extrapolated from bi-weekly Our world in Data dashboard (http://ourworldindata.org, accessed on November 25, 2022) and weekly reports of the Robert Koch Institute.

Human research participants

Policy information about studies involving human research participants and Sex and Gender in Research.

Reporting on sex and gender	Only data on sex were collected and are given in the manuscript. 684 particiants reported to be female, 722 reported to be male. We followed the guidance on sex and gender reporting because i) our research findings do not apply to only one sex, ii) in our study, sex of participants was determined based on self-report, and iii) the data are reported disaggregated for sex.
Population characteristics	During the time of sample collection (August and September 2022), 10,191 patients seeked medical treatment in the emergency departments. Of those patients, 1,411 (13.9%) were enrolled for study participation. Enrolled participants had a median age of 53 years (range 18-98, IQR: 35-69) with an overall balanced sex distribution (48.5% female; 51.3% male). 64.2% of the participants reported pre-conditions related most frequently to cardiovascular (52.3%) and neoplastic (24.4%) diseases. 13.6% of the participants reported drug immunosuppression at the time of sample collection.
Recruitment	Recruitment of participants and sample collection was conducted at five study sites in North Rhine-Westphalia, Germany (University Hospital of Cologne, University Hospital of Düsseldorf, University Hospital of Essen, University Hospital of Bonn, and University Hospital of Münster). Participation was offered to patients receiving medical treatment in emergency departments at one of the five study sites between August 8, 2022 and September 19, 2022. The study personnel recruited 1,411 participants in cooperation with the emergency department personnel. Patients were required to meet the following eligibility criteria in order to be enrolled as participants: i) only individuals aged ≥18 years were eligible, ii) participants had to be patients at the emergency department at one of the five study sites, iii) patients had to be able to consent to participate, iv) the ability to consent was furthermore checked by the study personnel with a special focus on the medication, pain and the exceptional emotional situation of the patients, and v) according to the assessment of the study personnel, participation in the study should not be a significant additional burden for the patients. As participants were recruited based on convenience sampling, self-selection bias of volunteers might be present. This might impact the external validity of the overall seroprevalence and we stated this in the discussion. However, we don't expect potential self-selection bias to have an impact on e.g., the correlation between the S-IgG levels and the serum neutralization.
Ethics oversight	All samples and data were obtained under protocols approved by the ethics committees of the Medical Faculty of the University of Cologne (22_1262), of the Medical Faculty of the University of Bonn (314/22), of the Medical Faculty of the University of Düsseldorf (2022-2072), of the Medical Faculty of the University of Essen (22-10838-BO), and of the Medical Faculty of the University of Münster (2022-490-b-S). All participants provided written informed consent. This study was registered as clinical trial (DRKS00029414).

Note that full information on the approval of the study protocol must also be provided in the manuscript.

Field-specific reporting

Please select the one below that is the best fit for your research. If you are not sure, read the appropriate sections before making your selection.

☒ Life sciences ☐ Behavioural & social sciences ☐ Ecological, evolutionary & environmental sciences

For a reference copy of the document with all sections, see nature.com/documents/nr-reporting-summary-flat.pdf

Life sciences study design

All studies must disclose on these points even when the disclosure is negative.

Sample size	The aim of this study was to determine point sero-prevalence during August and September 2022 at five study sites in North Rhine-Westphalia, Germany. We expecetd 50 patients per day per study site and expected 10-20% of all patients to be eligible for study participation. Power analysis with the function pwr.f2.test in the R package pwr showed that for detecting a small effect size F2 of 0.1 (roughly R2 model fit of 0.1) of a linear model with a power of 90% and significance Level 5%, we would need at least 229 to 235 individuals including all 12 (response= S-IgG) to 13 (including S-IgG as a feature) features. To further strengthen the analysis and because we aimed at a time period of 6-8 weeks, we enrolled 1,411 individuals in the study.
Data exclusions	Eligibility criteria for participation were i) only individuals aged ≥18 years were eligible, ii) participants had to be patients at the emergency department at one of the five study sites, iii) patients had to be able to consent to participate, iv) the ability to consent was furthermore checked by the study personnel with a special focus on the medication, pain and the exceptional emotional situation of the patients, and v) according to the assessment of the study personnel, participation in the study should not be a significant additional burden for the patients. Data of 17 participants were excluded due to insufficient blood volume for analysis. 1 participant has withdrawn consent after consenting and was therfore excluded.
Replication	For the pseudovirus neutralization assay, average inter-assay coefficient was determined as 18.09%, by testing 1,546 serum samples in duplicates on different plates and different days. Other assays besides the mentioned pseudovirus neutralization assay were not performed in duplicates. However, negative and positive percent agreement of the assays for S-and NC-IgG-detection was calculated with four different commercial assays at different study sites, respectively, as stated below and in the Methods part of the mansucript.

Randomization	Given the observational design of the study, randomization procedures were not applicable.
Blinding	Given the observational design of the study, blinding procedures were not applicable.

Reporting for specific materials, systems and methods

We require information from authors about some types of materials, experimental systems and methods used in many studies. Here, indicate whether each material, system or method listed is relevant to your study. If you are not sure if a list item applies to your research, read the appropriate section before selecting a response.

Materials & experimental systems

n/a	Involved in the study	
☐	☒	Antibodies
☐	☒	Eukaryotic cell lines
☒	☐	Palaeontology and archaeology
☒	☐	Animals and other organisms
☐	☒	Clinical data
☒	☐	Dual use research of concern

Methods

n/a	Involved in the study	
☒	☐	ChIP-seq
☒	☐	Flow cytometry
☒	☐	MRI-based neuroimaging

Antibodies

Antibodies used

For the neutralization assay, a SARS-CoV-2 neutralizing monoclonal antibody was used as run control (KV-Ab-188; R121-1F1, previously described by Vanshyllah at al. Cell Host Microbe 2022)

S-IgG were measured using DiaSorin's LIAISON® SARS-CoV-2 TrimericS chemiluminescence immunoassay (Cat#P/N311510) with the following cut-off values: negative < 33.8 BAU/ml and positive ≥ 33.8 BAU/ml.

NC-IgG were measured using the Euroimmun anti-SARS-CoV-2-NCP-ELISA (Cat#EI 2606-9601-2 G). S/CO values were interpreted as positive (S/CO ≥1.1), borderline (S/CO ≥ 0.8 - < 1.1), and negative (S/CO < 0.8).

Validation

Both assays were uses as per manufacturer's recommendations.

For S-IgG detection, positive percent agreement (PPA) and negative percent agreement (NPA) with i) Anti-SARS-CoV-2 QuantiVac IgG BAU (Euroimmun) (n = 502, measured at the study site in Düsseldorf) were 99.78% and 83.33%, with ii) DiaSorin's LIAISON® SARS-CoV-2 TrimericS chemiluminescence immunoassay (n = 185, measured at the study site in Essen) were 100% and 100%, with iii) Abbott's anti-SARS-CoV-2 IgG Quant II chemiluminescence microparticle assay (Alinity i) (n = 133, measured at the study site in Bonn) were 100% and 66.66% (n = 3), and with iv) Abbott's anti-SARS-CoV-2 IgG Quant II chemiluminescence microparticle assay (Alinity i) (n = 208, measured at the study site in Münster) were 99.5% and 100%.

For NC-IgG detection, PPA and NPA with i) Abbott's Architect SARS-CoV-2 IgG assay (n = 502, measured at the study site in Düsseldorf) were 97.6% and 94.85%, with ii) Abbott's Architect SARS-CoV-2 IgG assay (n = 185, measured at the study site in Essen) were 100% and 95.95%, with iii) Roche's Elecsys®-Assay (n = 133, measured at the study site in Bonn) were 56.86% and 100%, and with iv) Abbott's Architect SARS-CoV-2 IgG (n = 208, measured at the study site in Münster) were 83.05% and 98.92%.

The monoclonal antibody used as run control was previously described by Vanshyllah at al. (Cell Host Microbe. 2022).

Eukaryotic cell lines

Policy information about cell lines and Sex and Gender in Research

Cell line source(s)	HEK293T cells (ATCC, Cat#CRL-11268) and 293T-ACE2 (Jesse Bloom Lab; Crawford et al., 2020; BEI Resources, Cat#NR-52511).
Authentication	The cell lines were not authenticated.
Mycoplasma contamination	The cells were not tested for mycoplasma contamination.
Commonly misidentified lines (See ICLAC register)	The used cell lines are not commonly misidentified.

Clinical data

Policy information about clinical studies

All manuscripts should comply with the ICMJE guidelines for publication of clinical research and a completed CONSORT checklist must be included with all submissions.

Clinical trial registration	DRKS00029414

Study protocol	The protocol is available at DRKS00029414
Data collection	Data were collected at five study sites between August 8, 2022 and September 19, 2022.
Outcomes	The primary outcomes were the S-IgG levels (BAU/ml) and the vaccination status (according to German COVID-19 vaccination recommendation). The secondary outcomes were the neutralizing activities against Wuh01, BA.4/5 and BQ.1.1 (ID50).
